# Alberta Family Caregiver Strategy and Action Plan: Enhancing Integration Across Health and Social Care Systems

**DOI:** 10.3390/ijerph23010137

**Published:** 2026-01-22

**Authors:** Jasneet Parmar, Vivian Ewa, Andrew Karesa, Angie Grewal, Lesley Charles, Linda Powell, Josephine Amelio, Ginger Bitzer, Shannon Saunders, Darlene Schindel, Kimberly Shapkin, Charlotte Pooler, Frances Ross, Leeca Sonnema, Sanah Jowhari, Michelle N. Grinman, Cheryl Cameron, Arlene Huhn, Paige Murphy, Johnna Lowther, Cindy Sim, Suzette Brémault-Phillips, Sharon Anderson

**Affiliations:** 1 Department of Family Medicine, Faculty of Medicine & Dentistry, University of Alberta, Edmonton, AB T6G 2T4, Canada; jasneet.parmar@albertahealthservices.ca (J.P.); lesley.charles@albertahealthservices.ca (L.C.); bitzer@ualberta.ca (G.B.); leeca@ualberta.ca (L.S.); 2 Department of Family Medicine, University of Calgary, Calgary, AB T2N 4N1, Canada; vivian.ewa@albertahealthservices.ca; 3 blueBell Village Ltd., Edmonton, AB T6W 5B4, Canada; akaresa@bluebellvillage.ca; 4 Faculty of Nursing, University of Alberta, Edmonton, AB T6G 1C9, Canada; asangha@ualberta.ca (A.G.); pooler@ualberta.ca (C.P.); 5 Imagine Citizens Network, Calgary, AB T2P 4K9, Canada; lpowell1066@gmail.com; 6 Family Caregiver, University of Alberta, Edmonton, AB T6G 2T4, Canada; amelio@telus.net (J.A.); sanah.jowhari@gmail.com (S.J.); 7 Assisted Living Alberta, Edmonton, AB T5J 3E4, Canada; shannon.saunders2@assistedlivingalberta.ca; 8 DarleneSchindel.Com, Spruce Grove, AB T7X 3S4, Canada; darlene@darleneschindel.com; 9 Faculty of Nursing, University of Calgary, Calgary, AB T2N 1N4, Canada; kimberly.shapkin@ucalgary.ca; 10 FAR Strategic, Edmonton, AB T5R 2C7, Canada; fran@farstrategic.ca; 11 Cumming School of Medicine, University of Calgary, Calgary, AB T2N 4N1, Canada; michelle.grinman@albertahealthservices.ca; 12 Canadian Virtual Hospice, Winnipeg, MB R3L 2P4, Canada; cheryl@virtualhospice.ca; 13 Alzheimer Society of Alberta and Northwest Territories, Edmonton, AB T6H 1M4, Canada; ahuhn@alzheimer.ab.ca; 14 Alberta Health Services, Edmonton, AB T5J 3E4, Canada; pmwalker16@gmail.com; 15 Caregivers Alberta, Edmonton, AB T5B 1R1, Canada; jlowther@caregiversalberta.ca; 16 Team CarePal, Edmonton, AB T5J 1W8, Canada; cindy@teamcarepal.com; 17 Department of Occupational Therapy, Faculty of Rehabilitation Medicine, University of Alberta, Edmonton, AB T6G 2T4, Canada; suzette2@ualberta.ca

**Keywords:** family caregivers, caregiver-centered care, co-design, integrated care, implementation strategy, policy framework

## Abstract

**Highlights:**

**Public health relevance—How does this work relate to a public health issue?**

- Unpaid caregiving is a social determinant of health: caregiver well-being directly affects population health, system capacity, and the ability of older adults to remain safely at home.
- Across the evidence base, the primary precipitating factor for admission to hospital or long-term care is caregiver health breakdown or death, underscoring that sustaining caregiver well-being is a public health imperative.

**Public health significance—Why is this work of significance to public health?**

- This Strategy is one of the first to translate sound evidence, including that high-intensity caregiving is associated with poorer physical, mental, and economic outcomes, into an actionable provincial plan to prevent caregiver decline before crisis.
- By embedding triadic care, routine caregiver needs assessment, and integrated navigation, the Strategy addresses upstream drivers of hospitalization, institutionalization, and avoidable health system use.

**Public health implications—What are the key implications or messages for practitioners, policy makers and/or researchers in public health?**

- Practitioners and policymakers must treat caregiver identification, needs assessment, and partnership as core health care and safety practices, not optional add-ons; routine inclusion safeguards both caregivers’ health and the health of those they support.
- Researchers and policymakers should prioritize data systems, evaluation, and structural supports that reduce high-intensity caregiving burden—aligning with evidence that caregivers face inconsistent health outcomes, elevated financial strain, and major gaps in support.

**Abstract:**

Family caregivers provide up to 90% of care in Alberta’s communities and play an essential role in sustaining the province’s health and social care systems, yet they remain under-recognized and insufficiently supported. To address this gap, we co-designed the Alberta Family Caregiver Strategy and Action Plan (2024–2025), a provincial framework developed through participatory research and collective impact methods. Guided by principles of co-production, equity, and lived experience, the project engaged over 500 stakeholders, including caregivers, healthcare providers, educators, employers, and policymakers, through Phase 1 interviews (health/community leaders, *n* = 44; Family and Community Support Services (FCSS), *n* = 47; navigation experts, *n* = 9), Phase 2 co-design team consultations, and Phase 3 sector roundtables (*n* = 52). Using reflexive thematic analysis, we identified four foundational caregiver strategies, Recognition, Partnership, Needs Assessment, and Navigation, and four enabling conditions: Education, Workplace Supports, Policy and Research and Data Infrastructure. These elements were synthesized into an eight-priority Alberta Caregiver Strategy and Action Plan Framework, a practical way to connect validated priorities with coordinated, measurable implementation across settings. Participants emphasized four key enablers essential to making caregiver inclusion more feasible and sustainable: education, workplace supports, policy infrastructure, and research and evaluation. Findings highlight strong cross-sector consensus that caregiver inclusion must be embedded into routine practice, supported by consistent policy, and reinforced through provincial coordination with local adaptation. The Alberta Family Caregiver Strategy provides a practical, evidence-informed plan for transforming fragmented supports into a coherent, caregiver-inclusive ecosystem that strengthens both caregiver well-being and system sustainability.

## 1. Introduction

Over the past two decades, there has been growing recognition of the essential role that family caregivers play in sustaining health and social care systems. As early as 2007, Janice Keefe [1] warned that increased reliance on unpaid caregivers would be unsustainable without coordinated policy responses. Since then, the evidence has only strengthened: caregivers provide the majority of care in homes and communities, support system navigation, and maintain continuity of care, often bridging the gaps left by fragmented service delivery models [2,3,4].

Despite a wealth of research on effective caregiver interventions, translation into practice remains limited. Odom et al. [5,6] reported that although over 100 clinical trials showed positive outcomes for cancer caregivers, fewer than 12% of U.S. cancer centers had integrated these programs into routine care. The issue is not a lack of evidence, but a lack of structured implementation pathways, leadership, and policy levers [7,8,9,10]. This implementation gap is global, and Alberta is no exception.

Although caregiver strategies are increasingly articulated at national levels, implementation typically occurs at sub-national or regional levels where health and social service delivery is organized, funded, and coordinated. Alberta is presented here as a case study of a universal challenge: how to translate national caregiving commitments into actionable, measurable, and locally adaptable system change within a provincial jurisdiction. The strategy development process described in this paper therefore offers transferable insights for other regions seeking to operationalize caregiver partnership through shared infrastructure, cross-sector coordination, and implementation-focused policy levers.

Alberta’s caregivers provide between 75 and 90% of care in community settings and 15–40% in continuing care [11,12,13]. In 2018 alone, over one million Albertans contributed 647 million hours of unpaid care, the equivalent of more than 317,000 full-time jobs, valued at approximately $12 billion annually [14,15]. In contrast, Alberta’s formal health workforce in 2023 totaled 137,263 full-time people [16]. Despite outnumbering paid providers nearly seven to one, caregivers remain under-recognized, often serving as de facto system navigators, case managers, and care providers without formal training or support.

At the same time, the profile of caregivers is changing. With increasing longevity and rising complexity in the older adult population, caregiving responsibilities have shifted heavily onto workforce-aged adults [17,18]. Many fall within the sandwich generation, simultaneously caring for aging parents while supporting dependent children. This creates significant time, financial, and emotional strain, and amplifies the consequences of inadequate formal supports. Within the health and social care workforce itself, double-duty caregivers, paid providers who also deliver substantial unpaid care at home, are increasingly common [19,20,21]. In some continuing care settings in Alberta, more than half of staff also provide unpaid care at home [21]. These dual responsibilities heighten burnout risk and intensify the urgency for system-level solutions that recognize caregivers as essential partners, not invisible labor.

This invisibility has tangible consequences. Many caregivers face burnout, financial stress, and poor health outcomes. As Donna Wilson and colleagues [22] in their longitudinal study (weekly for six months) of Alberta Caregivers (*n* = 150) noted, they often lack access to mental health services, respite, and consistent community programs. These challenges demand timely, coordinated investment if Alberta’s care infrastructure is to remain sustainable.

Internationally, promising models are emerging. The 2022 U.S. RAISE National Strategy [23] outlines five priorities and over 150 actions for implementation. Eurocarers’ Strategy 2023 to 2030 [24] similarly emphasizes multi-level collaboration. In Canada, the Canadian Centre for Caregiving Excellence has co-designed a proposed National Caregiving Strategy [25], organized around five pillars: caregiver supports, education, financial aid, workforce development, and policy leadership [26]. The Canadian Centre for Caregiving Excellence “Caring in Canada” report found that over half of caregivers providing over five hours of weekly care experience financial strain, while 58% report chronic fatigue [27,28]. Meanwhile, 80% of paid long-term care providers have considered leaving the sector because of stress and poor working conditions.

Although the 2024 federal budget supports the development of a National Caregiving Strategy, health and social care remain constitutionally defined provincial responsibilities. Thus, Alberta’s Caregiver Strategy and Action Plan is not only timely, but also essential. While the national framework identifies shared priorities, Alberta’s Strategy translates these into actionable steps rooted in provincial contexts and system realities.

Caregivers in Alberta, like those across Canada and in most countries, remain the de facto system navigators [4,29,30]. They routinely bridge gaps between health, social, community, and housing systems, often acting as case managers without training or support. It is this navigation load and care-coordination burden, rather than hands-on care alone, that is strongly associated with caregiver distress, overwhelm, and burnout [31,32]. Although many supports and services already exist, families experience them as fragmented, disconnected, and difficult to access.

To address this, the Strategy emphasizes warm handoffs, shared navigation, and integrated planning across care settings. Warm handoffs, direct, relational transitions where responsibility is transferred *with* the caregiver rather than *to* them, help reduce fragmentation, clarify roles, and prevent caregivers from being left to coordinate alone [33,34,35,36]. Participants stressed that meaningful change requires co-production, where caregivers help shape, not simply receive, strategies, tools, and policies.

A core principle emerging across consultations was the importance of triadic conversations and triadic decision-making, structured interactions in which the provider, caregiver, and person receiving care participate together. Participants emphasized that without intentional triadic processes, caregivers are often relegated to the margins of care planning, even when they are responsible for implementing complex care at home. Embedding triadic approaches into assessments, consent discussions, transitions, and team-based planning was consistently described as foundational to caregiver partnership.

Crucially, the Strategy advances integration not only among providers, but also integration of caregivers (and patients) within care teams. Rather than being external to coordination processes, caregivers are positioned as informed partners whose insights, preferences, and capacities shape care planning, transitions, and follow-up. Enhanced integration therefore refers to:

1. Strengthening collaboration across health, social, community, and housing teams, and
2. Embedding patients and caregivers within those collaborative processes to ensure shared situational awareness, co-planning, and continuity.

This shift from a dyadic to a triadic model of care was consistently described by participants as essential to safety, communication, and continuity, particularly in complex care transitions.

Anchored in a collective impact model, the Strategy aims to foster coherence, continuity, and sustainability across sectors, ensuring that caregivers are recognized not as default navigators, but as partners supported by an integrated team-based system. Our team led the province-wide co-design process that developed Alberta’s Caregiver Strategy as a system-wide response to the implementation gap. Grounded in evidence and lived and living experience, we engaged caregivers, providers, health and community leaders, educators, employers, and policymakers. These partners brought diverse forms of experiential knowledge: many health and social care providers are, or have been, caregivers themselves, and many have personal experience of needing care across the life course. Recognizing that experiences of care and caregiving are widely shared, the co-design process worked from the understanding that one caregiver’s story often reflects broader health, social, and community care system realities.

This paper describes the co-design process (2024–2025) that informed the development of the Alberta Caregiver Strategy, alongside results from province-wide roundtables aimed at validating the Strategy’s priorities and identifying actionable opportunities. Through extensive engagement with diverse stakeholders, we identified four foundational caregiver strategies, recognition, partnership, needs assessment, and navigation; and the four enabling conditions: education, workplace supports, policy, and research and data infrastructure. Together, these form the backbone of the Alberta Caregiver Strategy and Action Plan.

## 2. Materials and Methods

### 2.1. Study Design

Our approach was grounded in co-design and participatory research methods, drawing from Thorne’s interpretive description [37,38] and collective impact approaches [39,40]. This method was selected because it aligns with our goal: to generate practical, actionable insights to support family caregivers within complex health and social care systems. We recognized from the outset that caregivers and their partners: healthcare providers, educators, employers, and community leaders needed to be engaged not only as participants, but as co-creators of the Alberta Caregiver Strategy.

Consistent with the collective impact framework [39,40], the co-design process incorporated the five core conditions. A common agenda emerged through Phase 1 interviews and was refined through Phase 2 co-design to define shared priorities for caregiver inclusion. Shared measurement was advanced through the development of strategy-linked implementation indicators (e.g., caregiver identification flags, needs assessment uptake, warm handoffs). Mutually reinforcing activities were represented through sector-specific roundtables identifying actions tailored to distinct roles while aligning with the same foundational directions. Continuous communication occurred through iterative engagement across phases, including co-design team consultations and cross-sector validation roundtables. Backbone support was provided by the research team and co-design infrastructure, which coordinated engagement, facilitated sense-making, and supported synthesis into a strategy and action framework.

Although stakeholders were invited based on their professional or organizational roles, participants brought multiple, overlapping identities and transferable experiential knowledge to the co-design process. Many providers and leaders were also family caregivers; many caregivers had experience working within health and social care; and participants’ insights were shaped by intersecting roles such as parent, daughter, employee, manager, or advocate. These intersectional experiences enriched the co-design process by bringing forward diverse forms of experiential and contextual knowledge beyond any single defined role.

We applied Braun and Clarke’s reflexive thematic analysis [41,42,43,44,45] to examine the qualitative data gathered through this process. This flexible yet rigorous approach allowed us to remain responsive to emerging themes while ensuring our interpretations were grounded in participants’ lived and living experiences.

### 2.2. Guiding Principles and Phases

From the beginning, we anchored the project in the values of inclusion, equity, and respect for lived experience. We knew that many individuals, especially caregivers and health, social, and community care providers, had previously shared their stories countless times, often with little tangible change. Our co-design principles should honor those contributions and ensure that stories would lead to solutions.

The project unfolded across three phases between May 2024 and September 2025:

1. Project governance and principle-setting,
2. Discovery phase through interviews and consultations, and
3. Synthesis into a comprehensive provincial strategy and action plan through sector-based roundtables.

All phases included guidance and refinement with our Caregiver-Centered Co-design Team. See Supplementary Table S1 for a detailed timeline of the co-design process.

### 2.3. Participants and Recruitment

#### 2.3.1. Eligibility Criteria

Eligibility criteria were guided by interpretive description methodology, which emphasizes the inclusion of participants with experiential and contextual knowledge relevant to the phenomenon of interest. Individuals were eligible to participate if they: (1) had lived and living experience as a family caregiver and/or professional, leadership, or system-level roles related to caregiver support within Alberta’s health, social, community, education, or employment systems; (2) were currently working in, or had recent experience with, Alberta-based systems relevant to caregiver support; (3) were able to participate meaningfully in interviews or group-based roundtables; and (4) provided informed consent.

Individuals were excluded if they did not have relevant experience or roles related to caregiving or caregiver support in Alberta, were unable to provide informed consent, or were unable to participate in interview or roundtable discussions. No additional demographic exclusion criteria were applied.

Eligibility was assessed by the research team during initial participant identification and invitation; and confirmed through informed consent procedures prior to participation.

Consistent with interpretive description methodology and participatory co-design principles, we used purposive and iterative snowball sampling to recruit participants whose experiential, professional, and system-level knowledge could inform practical and actionable solutions to support family caregivers within Alberta’s health, social, community, education, and employment systems. Recruitment was led by the research team, with guidance from the Caregiver-Centered Co-design Team and input from participants in early interviews and roundtables who identified additional individuals, sectors, or organizations relevant to the emerging analysis.

#### 2.3.2. Recruitment Procedures

Initial participant identification was informed by the research team’s long-standing engagement in Alberta-based caregiver consultations (2014, 2016, 2017), the development of the Caregiver-Centered Care Competency Framework (2018–2019), and the co-design, delivery, and evaluation of Caregiver-Centered Care Education. This sustained engagement provided access to a broad and diverse network of caregivers, healthcare providers, educators, employers, community leaders, and policy stakeholders across the province.

In keeping with interpretive description’s emphasis on variation and relevance, early recruitment priorities were further refined through 44 preliminary interviews with Alberta health and community system leaders and through ongoing consultation with the Caregiver-Centered Co-design Team. These processes helped identify priority sectors, care settings, populations, and geographic contexts where caregiver experiences and system challenges differed meaningfully and where practical insights were most needed. Recruitment intentionally sought both breadth and depth across care settings (acute, primary, emergency, home care, transitions, palliative care, assisted/supportive living, and long-term care), sectors (health, social, community, education, and workplaces), populations (pediatric, adult, and older adult care), and urban, rural, and Indigenous communities.

Once potential participants were identified, members of the research team organized individuals into sector- or role-specific cohorts and issued one-time email invitations describing the purpose of the study, the co-design approach, and expectations for participation. All invitations were sent directly by the research team. Partner organizations and individual participants supported recruitment by suggesting relevant sectors or individuals but did not recruit participants directly or manage enrolment.

#### 2.3.3. Snowball Sampling

Snowball sampling was used iteratively to enhance conceptual depth and ensure that emerging gaps in representation were addressed, consistent with interpretive description’s analytic logic. During interviews, co-design meetings, and roundtables, participants were invited to suggest additional individuals, roles, or groups whose perspectives could strengthen the analysis (e.g., “Have you talked to…?”). Suggested names or organizations were reviewed by the research team for alignment with study aims, and, where appropriate, the research team contacted potential participants directly by email.

Participants were not asked to forward invitations themselves, nor were referrals contacted without the research team’s involvement. Snowball sampling occurred in limited subsequent rounds rather than as a formal multi-wave process and was used to broaden cross-sector and geographic representation rather than to recruit within closed or homogeneous networks.

#### 2.3.4. Ethical Safeguards and Voluntariness

Recruitment and participation were grounded in principles of voluntariness, respect, and transparency. Email invitations were sent once, with no follow-up reminders, and participation or non-participation had no impact on professional relationships or future involvement. Informed consent materials and verbal introductions at the start of each interview or roundtable emphasized that participation was voluntary and that participants could choose how and what they wished to contribute.

Given the group-based nature of roundtables, participants were informed verbally and in writing that confidentiality could not be fully guaranteed and were asked not to share confidential or identifying information about others and to respect the privacy of group discussions. All interviews and roundtables were transcribed verbatim, and identifying information was removed during transcript cleaning prior to analysis. Only de-identified data were used in analysis and reporting.

#### 2.3.5. Participant Cohorts

Participants were recruited from four broad cohorts:

1. Health care providers, including acute, primary, emergency, home, palliative, continuing, and geriatric care, as well as pharmacy services;
2. Community and social service organizations, including Caregivers Alberta, Family and Community Support Services (FCSS), Indigenous organizations, seniors’ centers, and advocacy groups;
3. Education and training sectors, including post-secondary institutions, educators, and professional associations; and
4. Workplaces and employer organizations, including human resource professionals and caregiver advocacy representatives.

### 2.4. Data Collection

Data collection and analysis proceeded iteratively across three phases. Phase 1 generated an initial strategy pool from interviews; Phase 2 refined and organized these with the co-design team; Phase 3 validated and prioritized through sector roundtables. At each phase we conducted within-phase coding and then cross-phase synthesis to carry forward, refine, or retire candidate actions.

Phase 1 (July–September 2024): We conducted 44 interviews with health and social care providers, organizational leaders, and policymakers to map current caregiver supports and challenges. Realizing the critical role of FCSS programs, we subsequently conducted additional interviews with 47 FCSS providers and leaders. The FCSS mandate is to build resiliency, strengthen support networks, and prevent crisis by providing locally driven social supports before people require more intensive health or social services. We also spoke with nine groups of navigation experts, defined as individuals whose roles involve supporting or designing service navigation across health, social, and community systems (e.g., 211/811 operators, Help Seeker Technologies, primary care or community navigators, home care case managers, care transition coordinators, social workers/link workers involved in referrals, and system-level navigation leads involved in directory design and pathway development), to understand enablers and pain points in information sharing and cross-sector navigation. See semi-structured interview guide in Supplementary File S2.

Phase 2 (Bimonthly from September 2024 to September 2025 and ongoing): Our co-design team: comprising caregivers, providers, educators, and system leaders, participated in facilitated Zoom consultations. Using breakout rooms and shared documents, the team validated 18 emerging priority areas and worked to consolidate these into a draft Strategy framework.

Phase 3 (January–September 2025): We convened 52 sector-specific roundtables with a total of 371 participants. Each session included 2 to 12 participants and lasted approximately 60 min. We arranged one-on-one interviews for individuals with scheduling constraints to ensure inclusive participation. Tailored facilitation guides were used to explore alignment with the draft Alberta Caregiver Strategy, surface sector-specific needs, and identify actionable next steps. See Supplementary Table S1 for a detailed timeline of the co-design process.

### 2.5. Data Analysis

We analyzed data using reflexive thematic analysis within a constructivist, interpretive description frame. Consistent with Braun and Clarke’s [41,42,43,44,45] approach, we understood themes as actively constructed through researcher engagement with the data, rather than passively “discovered.” Reflexivity was integral throughout: our positionalities, as researchers, practitioners, and caregivers, shaped how we interpreted the data, the questions we asked of it, and the meanings we constructed.

Analysis proceeded phase-by-phase and cumulatively:

- Phase 1 (Interviews): inductive coding to construct early patterns of meaning and candidate strategic areas.
- Phase 2 (Co-design): abductive refinement through collaborative sense-making, that is constructing core strategies, supporting strategies, and strategic actions.
- Phase 3 (Roundtables): iterative, deductive–inductive engagement with the draft framework to further shape, nuance, and complicate emerging interpretations.

After each phase, we performed cross-phase synthesis to update the developing strategy set and document changes (added, merged, reworded, or retired actions).

#### 2.5.1. Identifying the Initial List of Strategies

In Phase 1, our analysis focused on constructing an initial set of strategies, defined as actionable policies, programs, or interventions with potential to strengthen caregiver recognition, support, and inclusion. Insights were inductively coded and clustered into preliminary priority areas.

In Phase 2, these early constructions were collaboratively refined with our co-design team. Together, we organized the strategies into:

- Core strategies: What actions must be taken to support caregivers.
- Supporting strategies: How these actions can be implemented across settings.
- Strategic actions: Tactical, context-specific steps or enablers.

This collective interpretive work expanded the Strategy from 18 initial priorities to 26 strategic actions organized under core and supporting strategies.

#### 2.5.2. Validating and Strengthening the Strategy

In Phase 3, sector-specific roundtables were used to further shape and refine the developing strategy. Transcripts were coded to examine how participants’ perspectives aligned with, complicated, or challenged emerging strategic elements, using the following coding categories:

- Explicit alignments: direct statements of support or proposed additions.
- Interpretive alignment: patterns of meaning that resonated with strategic directions.
- Divergences: sector-specific tensions, competing priorities, or contextual constraints.

We synthesized interpretations at each roundtable, collated findings by cohort (e.g., FCSS, seniors’ centers, home care providers), and then reviewed all cohorts together to identify:

- Cross-sector priorities with broad relevance.
- Sector-specific needs reflecting local contexts, and.
- Opportunities for collective action that could enhance system-wide impact.

#### 2.5.3. Ensuring Rigor and Relevance

Throughout the analysis, we emphasized interpretive rigor and contextual relevance by integrating reflexive practices and ongoing engagement with participants and co-design partners. Consistent with reflexive thematic analysis, rigor was supported through reflexive memoing, team-based interpretive dialogs, and explicit attention to how our positionalities shaped the meanings we constructed from the data. Preliminary analytic constructions were shared with participants and partners, not to “verify” themes, but to enhance their resonance, usefulness, and contextual fit across diverse settings.

This participatory and layered analytical process allowed us to build a Strategy grounded in both the systemic realities of Alberta’s care infrastructure and the lived and living experience of caregivers. The result is a coherent, evidence-informed framework that reflects both consensus and complexity, capturing what matters most to caregivers and those who support them.

## 3. Results

### 3.1. Sample Description

A total of 371 participants took part in 52 roundtables conducted across Alberta between May 2024 and September 2025. Participants represented a broad range of stakeholder groups involved in supporting family caregivers, including health care providers, community and social service organizations, education and training sectors, workplace and employer organizations, policy and system leaders, and condition- and population-specific advocacy organizations. This breadth reflects the study’s interpretive description and co-design orientation, which prioritizes diverse experiential and system-level perspectives to inform practical solutions. Table 1 summarizes participant characteristics and stakeholder representation.

### 3.2. Care Settings and Populations Represented

Participants contributed perspectives from across the full continuum of care. Health care representation included acute and intensive care, primary care, emergency care, home care, palliative care, rehabilitation and restorative care, mental health and addictions, transplant services, pharmacy, assisted and supportive living, and long-term care. Community-based perspectives included Family and Community Support Services (FCSS), seniors’ centers, age-friendly initiatives, caregiver organizations, and Indigenous community organizations.

The sample reflected care experiences across the lifespan, including pediatric, adult, and older adult populations, and included perspectives from urban, rural, and Indigenous communities across Alberta. Participants frequently held multiple, intersecting roles (e.g., clinician and family caregiver; educator and practitioner), which enriched the analysis by bringing forward integrated insights spanning lived experience, professional practice, and system navigation. See Supplementary File S3: Distinctive angles by cohort (what each brings).

### 3.3. Findings Overview: Shared Priorities and Practical Actions

To reflect the breadth, depth, and consistency of perspectives across the 52 roundtables, the findings are organized in two complementary parts. Together, they illustrate strong cross-sectoral alignment alongside contextual nuance, consistent with the study’s interpretive description and co-design approach.

- Section 1 confirms that the Alberta Caregiver Strategy’s core directions are broadly supported across sectors and settings, drawing on participants’ narratives to illustrate convergence as well as variation in how challenges are experienced and addressed.
- Section 2 highlights the strongest cross-sectoral points of agreement by identifying a set of practical actions that participants consistently prioritized as high-impact and feasible for implementation.

Taken together, these findings lay the groundwork for a coordinated, caregiver-inclusive approach to health, social, community, education, and workplace system transformation in Alberta.

### 3.4. Section 1: Confirmation of Strategy Directions

**Figure 1:**
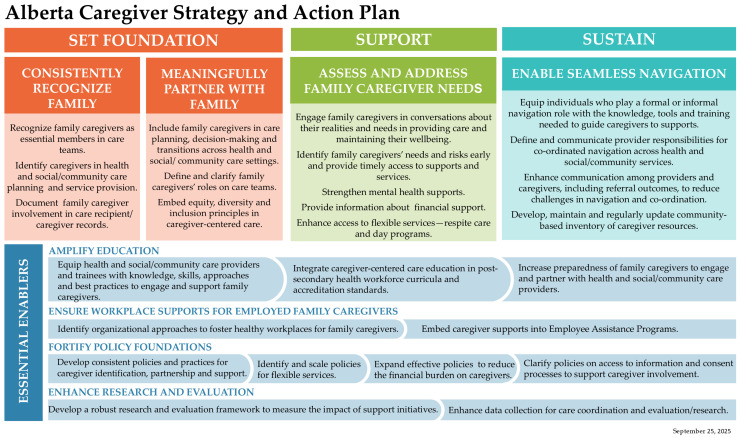
Alberta Caregiver Strategy and Action Plan.

This section demonstrates widespread alignment with the Alberta Caregiver Strategy’s nine components—four foundational directions and five enabling strategies—across diverse sectors and care contexts. From acute and intensive care teams to rural FCSS programs, from Indigenous organizations to employer and education sectors, participants described common system gaps and articulated shared priorities for change. The subsections that follow highlight each strategy direction using illustrative quotes that reflect both consensus and contextual nuance (see Figure 1 and Table 1).

#### 3.4.1. Recognition: Naming Caregivers in Health, Social, and Community Care Systems

Recognition was consistently described as the “entry point” to all other strategy directions. Without formal identification, caregivers remain invisible in care records, decision-making processes, and policy frameworks. Caregivers reported that this invisibility eroded their legitimacy and undermined trust in care relationships. Providers echoed this concern, noting that the absence of clear caregiver identification left teams uncertain about how, when, or with whom to engage, particularly during care transitions and periods of heightened risk.

#### 3.4.2. Partnership: Moving from Helper to Collaborator

Participants emphasized that caregivers are already performing substantial care work, including administering medications, coordinating appointments, monitoring symptoms, and managing crises. What they sought was not additional responsibility, but recognition and support as partners in care. Providers and policymakers highlighted that partnership must be embedded in everyday clinical and organizational processes, particularly consent discussions, care transitions, and team-based planning, and must be co-developed with caregivers to ensure relevance and sustainability.

**Figure 2:**
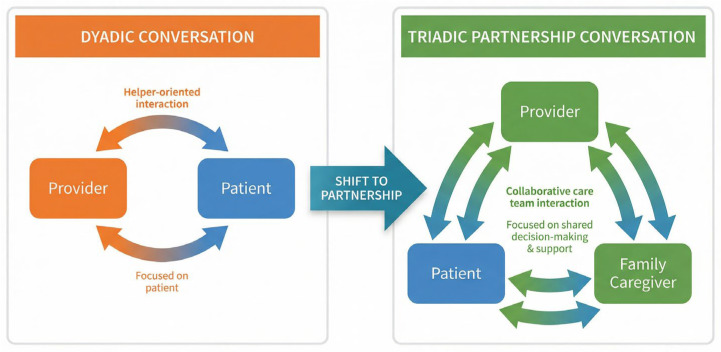
From Dyadic to Triadic Care.

Across sectors, participants stressed that partnership is operationalized through triadic conversations and decision-making, in which the caregiver’s voice is included alongside that of the person receiving care and the provider. This triadic approach was viewed as essential to improving safety, continuity, and shared understanding. To clarify the structural shift described by participants, Figure 2 illustrates the transition from dyadic (provider–patient) interactions to triadic (provider–patient–caregiver) partnership and decision-making.

The triadic care model positions the caregiver as an active partner alongside the person receiving care and the provider(s), enabling shared situational awareness, co-planning, and continuity across care transitions. The dyadic model illustrates how caregivers are often excluded from routine communication and decision-making despite being responsible for implementing care plans outside formal settings.

#### 3.4.3. Access to Information and Privacy Clarity: Clarifying the Circle of Care

Participants across sectors emphasized that misunderstandings, rather than privacy legislation itself—often impede effective communication with caregivers. Many noted that privacy requirements are frequently interpreted in overly restrictive ways, resulting in caregivers being excluded from essential care discussions, discharge planning, and safety planning. Participants emphasized that such exclusions can increase risk and harm, particularly when caregivers are responsible for implementing care plans at home.

To address this gap, participants called for clear, plain-language consent practices; standardized guidance for triadic care conversations; and system-level messaging that affirms appropriate information sharing with caregivers within the circle of care as both permitted and necessary for safe, effective, and equitable care.

#### 3.4.4. Needs Assessment: Recognizing Risks Early

Caregivers often receive support only once they are already in crisis. Providers acknowledged that they lack consistent tools to recognize caregiver distress before it escalates. Participants strongly endorsed the use of standardized screening tools, such as the Carer Support Needs Assessment Tool (CSNAT) Intervention, and emphasized the importance of equipping providers to initiate proactive, early, and ongoing conversations with caregivers about their needs.

#### 3.4.5. Navigation: A System Burden Carried by Family Caregivers

Navigation was one of the most frequently cited pain points. Caregivers described the system as fragmented, overwhelming, and reliant on their own persistence to “Google their way through.” Providers echoed this concern, with many unsure of how or where to refer caregivers. Rather than building new structures, participants called for better integration of existing platforms (e.g., 211 Alberta, Primary Care or community navigators, Home Care Case Managers, FCSS, social prescribers/link workers) and a shared responsibility for navigation across sectors.

#### 3.4.6. Education: Preparing Providers and Caregivers

Participants highlighted a dual need for education. Providers require foundational training in caregiver partnership embedded into onboarding, simulation, and accreditation, while caregivers need tailored learning supports to build confidence, clarify roles, and prepare for transitions. Across groups, participants emphasized that education must be timely, culturally responsive, and embedded across the care continuum.

#### 3.4.7. Workplace Supports: Sustaining the Dual Role

Participants described the strain experienced by employed caregivers attempting to balance work and care responsibilities, including exhaustion, stigma, and inflexible work environments. Employers and human resource professionals reported a lack of guidance and tools to support caregiving staff. Many called for caregiver-friendly policies to be embedded within human resource systems and framed caregiving within psychological safety and workforce retention strategies.

#### 3.4.8. Policy and Research Infrastructure: Creating Foundations for Change

Participants cautioned that without policy infrastructure, caregiver supports would continue to rely on local champions rather than systemic design. They emphasized the need for mandates, standards, and flexible funding models, alongside evaluation frameworks that measure outcomes caregivers value, such as inclusion, access, and continuity. See Table 2: Cross-Cutting Strategies with Quotes.

### 3.5. Section 2: Cross-Sector Actions for Implementation

While all strategy directions were endorsed as important, a smaller set of actions consistently emerged as high-impact and cross-cutting. These actions were repeatedly identified by participants across all sectors as practical, near-term opportunities to catalyze system-wide change. They bridge health, social, community, education, and workplace systems, as well as urban and rural contexts and population-specific needs.

The following nine actions were consistently prioritized across sectors:

1. Make caregivers visible in the record and the room: Electronic Medical Record flags, routine caregiver identification, and role clarity across care settings.
2. Education for providers: Caregiver partnership training embedded in onboarding, curricula, and continuing education.
3. Education for caregivers: Learning supports based on caregiver needs, care trajectories, and cultural context.
4. Assess and address caregiver needs early and routinely: Use of screening tools (e.g., CSNAT), proactive planning, and referral systems.
5. Flexible caregiver supports: Mental health services, overnight respite, short-stay options, practical supports, and bereavement care.
6. Navigation infrastructure that connects, not fragments: Warm handoffs, relational navigation, and system integration (e.g., FCSS, 211, clinic navigators).
7. Workplace and financial security: Employer engagement, benefits integration, and policy levers (e.g., pensions, tax supports).
8. Policy and data backbone: Mandates, metrics (e.g., flag usage, burnout rates), and evaluation of caregiver outcomes.
9. Consent and privacy clarity for triadic care: Plain-language tools and shared frameworks to include caregivers ethically and legally. See Table 3: Cross-Sector Implementation Priorities and Levers for Action.

### 3.6. Toward an Alberta Caregiver Strategy and Action Plan

Building on these cross-sector actions, findings were synthesized into the Alberta Caregiver Strategy and Action Plan. This framework translates insights from 52 roundtables and over 100 interviews into a focused set of provincial priorities aligned with system readiness and policy levers (see Table 4 Strategic Action Priorities).

### 3.7. Interpreting the Priorities

Taken together, these priorities provide a coherent, evidence-informed direction for Alberta’s next phase of action. Each builds upon the foundational strategies of Recognition, Partnership, Needs Assessment, and Navigation and is reinforced by the enabling conditions of Education, Workplace Supports, Policy, and Research. Rather than advancing isolated initiatives, the Alberta Caregiver Strategy emphasizes integration, shared accountability, and collective learning. In the following discussion, we situate these findings within broader national and international contexts, and implications for sustained implementation are examined.

## 4. Discussion

Our findings echo what has been described internationally: caregivers are indispensable to system sustainability but remain under-recognized, under-supported, and poorly integrated. What distinguishes Alberta’s consultations is the strong, cross-sector consensus not only on what needs to change but on how to begin. Participants repeatedly emphasized that advancing caregiver partnership requires normalizing triadic conversations and triadic decision-making as routine elements of care.

Recognition, partnership, needs assessment, and navigation emerged as foundational directions, while education, workplace supports, policy, and research provide the enabling conditions for sustained change. Together, these elements are operationalized through the eight strategic priorities outlined in the Alberta Caregiver Strategy’s Strategic Action Framework. These priorities, ranging from caregiver identification and needs assessment to education, workplace culture, and system integration, represent actionable, co-produced steps that stakeholders across Alberta are ready to implement.

### 4.1. Situating the Alberta Caregiver Strategy and Action Plan-Strategic Framework in Context

In many ways, Alberta’s Strategy mirrors other caregiver strategies. The U.S. RAISE National Strategy [23], Eurocarers Strategy to 2030 [24], and the Canadian Centre for Caregiving Excellence’s National Caregiving Strategy [25] all call for awareness, partnership, service supports, workplace accommodations, and research. This alignment underscores that caregiving challenges are universal.

However, Alberta’s Strategy advances this agenda by situating caregiver support within a collective impact and implementation framework, one that integrates national directions with Alberta’s provincial rural and urban contexts. It also explicitly embeds triadic care processes, an element less visible in earlier national strategies but central to Alberta stakeholders’ definitions of safe, relational, and integrated care. It builds on earlier Canadian policy work: Sinha et al. [46,47] called for proactive supports such as respite and income security, while the Canadian Centre for Caregiving Excellence [28] described caregivers as the “unseen and unacknowledged foundation upon which our health care, social services and disability supports systems are built.” The Alberta Strategy extends these calls by providing actionable mechanisms, shared data systems, education pathways, and navigation infrastructure to translate recognition into measurable change within provincial responsibilities.

### 4.2. Why Hasn’t This Worked Before?

Previous efforts often faltered because they relied on fragmented initiatives, short-term funding, or the efforts of individual champions [48,49]. Supports were designed in silos, leaving caregivers to piece together their own networks of care. Our consultations confirmed that this fragmentation persists across sectors. The Alberta Strategy addresses this by creating shared structures for accountability, embedding caregiver identification, assessment, and partnership practices into existing systems rather than adding new standalone programs.

A recurrent theme across our consultations was the system’s overreliance on individual champions to carry caregiver support initiatives forward. Participants stressed that without formal mandates, policy infrastructure, and stable funding mechanisms, even highly successful innovations remain vulnerable to staff turnover, burnout, and shifting priorities. This challenge is not unique to caregiver support work; it reflects a broader pattern across health and social systems, where change often depends on motivated individuals rather than embedded structures. Stakeholders consistently emphasized that sustained progress requires moving beyond champion-driven efforts toward systematized, policy-backed practices that persist regardless of local leadership changes.

### 4.3. What Might Make It Different This Time

We believe the Alberta Caregiver Strategy can succeed where earlier efforts stalled because it:

- Emphasizes integration. Linking health, social, and community systems through shared navigation, documentation, and evaluation frameworks.
- Emphasizes co-production. Engaging caregivers alongside providers, leaders, and policymakers in design, implementation, and evaluation.
- Balances provincial direction with local adaptation. Ensuring coherence and equity while allowing regional flexibility.
- Focuses on implementation tools. Providing concrete mechanisms such as toolkits, policy templates, and communities of practice to operationalize change.

Embedding triadic care as a standard practice was viewed as a critical lever for ensuring that partnership is enacted consistently rather than depending on individual practitioners.

### 4.4. The Way Forward

The message from our consultations is clear: Alberta already possesses many of the programs and supports caregivers need, but they are disconnected. The Alberta Caregiver Strategy and Action Plan- Strategic Framework offers a co-produced roadmap to align these components, through actions that are coordinated provincially, adapted regionally, and implemented locally.

If implemented successfully, caregivers will no longer be invisible or left to navigate systems alone. They will be recognized, supported, and engaged as partners in care, with equitable access to the supports needed to sustain their well-being. In doing so, Alberta can lead the way in building an integrated care ecosystem that shares responsibility across caregivers, providers, leaders, and policymakers.

Throughout the Alberta Caregiver Strategy and Action Plan- Strategic Framework, we emphasize that toolkits, practices, and policies must be co-developed at local, regional, and provincial levels. Programs such as social prescribing and community-based seniors’ supports should not remain isolated initiatives but should operate within a connected ecosystem that supports caregivers through coordinated, multi-level systems.

The Alberta Caregiver Strategy and Action Plan- Strategic Framework rests on a dual imperative:

- Shared provincial direction to ensure coherence and equity.
- Context-sensitive local adaptation to ensure relevance and effectiveness.

Toolkits are not end-products but living resources, developed collaboratively to meet diverse community needs while aligning with system-wide priorities. Similarly, policies must be established provincially but implemented regionally and refined locally to ensure accessibility and sustainability.

### 4.5. Strengths and Limitations

This study has several strengths. First, it drew on a large, province-wide and cross-sectoral co-design process, engaging 371 participants across 52 roundtables spanning health care, community and social services, education and training, workplaces and employers, policy and system leadership, and condition- and population-specific advocacy organizations. This breadth enabled the identification of shared priorities across care settings and contexts, while also capturing sector-specific nuance. Second, the interpretive description approach supported an applied, practice-oriented analysis focused on generating actionable insights for system improvement. Third, using Braun and Clarke’s reflexive thematic analysis enabled interpretive depth while remaining responsive to emerging patterns across diverse stakeholder perspectives. Finally, the co-design orientation, including ongoing guidance from the Caregiver-Centered Co-design Team, strengthened relevance and ensured that findings were grounded in lived and living experience as well as frontline realities.

Several limitations should be considered. Recruitment relied on purposive and iterative snowball sampling, informed by existing provincial networks and stakeholder recommendations. While this approach was appropriate for capturing information-rich perspectives and identifying practical implementation opportunities, it may have introduced network bias and may underrepresent individuals and communities less connected to established caregiver and service networks. In addition, the roundtable format supported cross-sector dialog but limited confidentiality and may have influenced what participants were willing to disclose in a group setting. We mitigated this through informed consent processes, reminders about confidentiality limits, and removal of identifying information during transcript cleaning; however, group dynamics and power differentials may still have shaped discussion. Finally, participant characteristics were documented primarily by sector and role rather than detailed individual demographics. This reflects the study’s system-oriented focus but limits the ability to assess demographic representativeness and may obscure differences in experiences related to factors such as age, gender, ethnicity, and socioeconomic position. Future work should examine how strategy priorities and implementation experiences vary across diverse caregiver populations and communities.

### 4.6. Future Prospects

The Alberta Caregiver Strategy and Action Plan–Strategic Framework provides a practical foundation for coordinated implementation; however, its impact will depend on how effectively priorities are operationalized across settings. Several next steps are particularly promising. First, implementation efforts should focus on establishing shared infrastructure for caregiver identification, routine needs assessment, and navigation pathways across sectors, supported by plain-language consent and privacy guidance that enables triadic care in practice. Second, education and workforce strategies should embed caregiver partnership competencies across training pathways and onboarding, including for medicine, nursing, allied health, healthcare aides, and personal support workers, alongside accessible learning supports for caregivers themselves.

Third, workplace and policy levers will be critical to sustain change. Employer-facing tools and standards, caregiver-friendly workplace policies, and attention to financial security can reduce strain for employed caregivers and support retention across the health and social care workforce. Finally, a research and evaluation agenda is needed to monitor implementation and outcomes that caregivers value, such as feeling recognized, included in decision-making, supported during transitions, and able to sustain their role without harm to health or livelihood. Early evaluation priorities could include monitoring caregiver identification practices, uptake of needs assessment processes, accessibility of navigation supports, and measures of caregiver stress and well-being. Ongoing co-development with caregivers and diverse communities—including rural, remote, and Indigenous contexts—will be essential to ensure that implementation remains equitable, culturally responsive, and locally relevant.

## 5. Conclusions

The Alberta Caregiver Strategy and Action Plan–Strategic Framework charts a path toward system-wide integration of caregiver supports by aligning provincial priorities with local implementation capacity. Through its eight strategic priorities, it provides a coherent, co-produced framework that connects policy, practice, and lived experience.

By emphasizing actionable tools, shared infrastructure, and cross-sector collaboration, Alberta is well positioned to advance a caregiver-inclusive care system that bridges traditional divides between health and social services. Sustaining caregivers ultimately requires reimagining care itself—not as a patchwork of disconnected programs, but as an integrated continuum of supports that begins in the community and is reinforced across local, regional, and provincial systems.

## Figures and Tables

**Table 1:** Descriptive Profile of Participants and Stakeholder Representation.

Characteristic	Description
Total participants (N)	371
Total roundtables conducted	52
Study period	May 2024–September 2025
Geographic scope	Province-wide (urban, rural, and Indigenous communities across Alberta)
**Stakeholder and Sector Representation**	
**Stakeholder group**	**Included sectors/roles**
Health care providers and clinical teams	Acute care, intensive care, primary care, emergency care, palliative care, rehabilitation/restorative care, mental health and addictions, transplant services, home care, continuing care (assisted/supportive living and long-term care), pharmacy
Community and social service organizations	Family and Community Support Services (FCSS), seniors’ centers, age-friendly initiatives, community-based caregiver organizations, Indigenous community organizations
Education and training sectors	Post-secondary educators and trainers in medicine, nursing, allied health, healthcare aides, and personal support workers; professional associations
Workplaces and employer organizations	Human resource professionals, employer representatives, workplace caregiver advocates
Policy, system, and organizational leaders	Health system leaders, community and municipal leaders, provincial representatives
Condition- and population-specific advocacy organizations	Adult and pediatric disease-based organizations, transplant-related organizations, mental health and addictions advocacy groups

**Table 2:** Cross-Cutting Strategies with Quotes.

Foundational Strategy/Theme	Summary of Evidence and Insights	Illustrative Quotes (Stakeholder)
Recognition	Caregivers must be formally identified and documented in care plans and Electronic Medical Records (EMRs) to ensure visibility and legitimacy.	“Right now, if you look in the chart, the caregiver doesn’t exist.”—Acute Care
		“We assume it’s next of kin, but the real caregiver might be someone else entirely.”—Seniors’ Centre
Partnership	Caregivers already perform complex care tasks and want to be acknowledged as collaborators rather than helpers.	“If we don’t teach triadic care, students won’t learn to partner with families.”—Educator “Partnership has to be built into transitions, not added on at discharge.”—Primary Care
Needs Assessment	Supports often come too late; early, standardized tools (e.g., CSNAT) are needed to identify caregiver stress and risk.	“We’re treating the patient, but the family is already falling apart.”—Geriatric Psychiatry “If you ask the caregiver at the start, you can plan supports before they burn out.”—FCSS
Navigation	System fragmentation leaves caregivers to coordinate care alone; they need warm handoffs and relational navigation.	“A list isn’t navigation—people need someone to walk it with them.”—Seniors’ Centre “Even I work in the system and can’t find help for my own parents.”—Provider
Education	Dual need: provider education in caregiver partnership and accessible learning for caregivers themselves.	“Microlearning is what busy clinicians will actually use.”—Primary Care “Families need training, not just pamphlets.”—Transplant Roundtable
Workplace Supports	Employed caregivers experience burnout and stigma; employers lack structured policies and guidance.	“Human Resources (HR) needs to look at caregiving the way they look at maternity leave—predictable and inevitable.”—HR Leader
Policy and Research Infrastructure	Without mandates and data, caregiver practices remain voluntary; stronger policy and measurement frameworks are required.	“We can’t improve what we don’t measure.”—Geriatric Medicine
		“Navigation and respite are stuck as pilots—we need policy that locks them in.” —Primary Care

**Table 3:** Cross-Sector Implementation Priorities and Levers for Action.

Priority Area	Implementation Focus/Lever	Illustrative Example or Early Metric
1. Caregiver Identification & Documentation	Embed caregiver fields and flags in EMRs and care plans through shared policy and data standards.	Percentage of records with caregiver flag; standardized fields across Connect Care and community systems.
2. Routine Needs Assessment and Response	Integrate brief screening (e.g., CSNAT) at intake and transitions; link results to referral workflows.	Proportion of caregivers screened; completed referral and follow-up rates.
3. Integrated Navigation Supports	Coordinate 211, FCSS or community social prescribers, and Primary Care navigators via warm-handoff protocols and shared directories.	Number of documented warm handoffs; frequency of shared navigation-directory use.
4. Provider and Caregiver Education	Embed caregiver-centered modules in curricula, onboarding, and public learning.	Programs including caregiver-centered care education modules; learner completion and confidence rates.
5. Flexible Caregiver Supports	Expand respite, day, and mental health services; address overnight and bereavement needs.	New respite or short-stay spaces created; uptake of counseling supports.
6. Workplace and Financial Security	Implement caregiver-friendly HR policies, flexible leave, and benefit integration.	Organizations adopting caregiver policies; employee self-identification rates.
7. Policy and Data Backbone	Establish provincial mandates, metrics, and dashboards to monitor caregiver outcomes.	Annual caregiver-outcome dashboard; policy adoption and evaluation coverage.
8. Consent and Privacy Clarity for Triadic Care	Develop plain-language tools and scripts for ethical caregiver inclusion in decision-making.	Teams using standardized consent scripts; staff-reported confidence levels.

**Table 4:** Strategic Action Priorities.

Priority	Example Outcomes
1. Mobilize and apply strategy knowledge in policy and practice.	Increased cross-sector awareness of caregivers as partners in care • Accelerated adoption of caregiver-inclusive practices at individual, organizational, and system levels
2. Standardize identification and documentation of family caregivers across systems.	Consistent Electronic Medical Record/care-plan fields to recognize the caregiver role • Improved coordination and information continuity between settings and providers
3. Implement routine caregiver needs assessment and response pathways.	Brief, validated screening at intake and key transitions • Clear referral and follow-up processes proportional to identified risk
4. Strengthen integrated navigation supports.	Formalized warm handoffs and relational navigation within/between sectors • Connected assets (e.g., FCSS, PCNs, 211/CIE) into coherent pathways
5. Embed caregiver-centered education across learning and service environments.	Caregiver partnership and triadic care in curricula, onboarding, simulation, and accreditation • Microlearning for just-in-time practice support
6. Foster caregiver-friendly and psychologically safe workplaces.	Normalized flexible arrangements and supervisor practices that support employed caregivers • Aligned benefits and policies to reduce financial strain and turnover
7. Enhance policy and financing levers for basic and flexible supports.	Sustainable funding for respite, mental health, and practical supports • Metrics and evaluation guiding resource allocation toward proven interventions
8. Equip caregivers to enact their partnership role.	Culturally responsive, trajectory-aligned learning and preparedness tools Plain-language consent/privacy guidance enabling shared decision-making

## Data Availability

The datasets used and/or analyzed during the current study are available from the corresponding author on reasonable request.

## Supplementary Materials

The following supporting information can be downloaded at: https://www.mdpi.com/article/10.3390/ijerph23010137/s1, Supplementary File S1: Supplementary Table S1. Co-Design Process for the Alberta Family Caregiver Strategy & Action Plan (May 2024–April 2025); Supplementary File S2: Interview Guide for Phase 1 Introductory Interviews. Supplementary File S3: Distinctive angles by cohort (what each brings).
