# Supplementary material for: Alberta Family Caregiver Strategy and Action Plan: Enhancing Integration Across Health and Social Care Systems
Source: Int J Environ Res Public Health. 2026 Jan 22;23(1):137. doi: 10.3390/ijerph23010137 (PMC12840734; doi:10.3390/ijerph23010137)
Supplement: Supplementary file 1 [file ijerph-23-00137-s001.zip › ijerph-4046840-supplementary.pdf]

Supplementary Table S1. Co-Design Process for the Alberta Family Caregiver Strategy & Action Plan (May 2024 – April 2025)

Phase	Activities	Outputs	Timeframe
Phase 1: Project Planning and Foundations	• Established governance structure and guiding principles<br>• Transitioned co-design team from earlier caregiver-centered education projects to strategy co-production<br>• Confirmed scope, values, and goals of the Strategy	• Governance framework<br>• Guiding principles<br>• Shared vision for caregiver-centered strategy development	May–June 2024
Phase 2: Draft Framework Co-Design	• Conducted 44 semi-structured interviews with health, social, and community providers, leaders, and policymakers<br>• Conducted 47 additional interviews with Family and Community Support Services (FCSS) staff and leaders and 9 with navigation experts<br>• Identified barriers, current supports, successes, and gaps	• Thematic synthesis of barriers and supports<br>• 18 initial priority areas for caregiver support	July–September 2024
Co-Design Team Consultations	• Convened Zoom meetings with caregivers, providers, policymakers, educators, researchers<br>• Used breakout groups to review interview findings<br>• Validated and refined emerging themes	• 18 potential priorities consolidated into draft primary and supporting strategies<br>• Guiding principles co-developed and affirmed	September–November 2024
Phase 3: Strategy Map Roundtables	• Held 52 sector-specific roundtables with 317 participants across health, community, education, and workplace cohorts<br>• Facilitated 60–90-minute discussions using semi-structured guides tailored to sector context<br>• Participants reviewed draft strategies, identified sector-specific priorities, and suggested actions	• Sector-specific insights and priorities<br>• Cross-cohort synthesis of shared strategies<br>• Draft Alberta Caregiver Strategy & Action Plan	January–September 2025
Phase 4: Launch	• Synthesized findings across all phases<br>• Developed integrated Strategy and Action Plan<br>• Prepared for dissemination and implementation	• Finalized Strategy Map (primary/supporting strategies, guiding principles)	October 2025–April 2026 (planned)

Supplementary File S2 Interview Guide for Phase 1 Introductory Interviews

Welcome

Hello and thank you for joining us today. My name is [name], and I am [title] with Dr. Jasneet Parmar's Caregiver Centered Care Team at the University of Alberta Our Caregiver-Centered Care Initiative is designed to support health, and community care providers to provide person-centered care to family caregivers.

We have funding from Alberta Health to co-design an Alberta Caregiver Strategy and Action Plan. This strategy aims to innovate integrating health and community services to build a better system to support family caregivers

We reached out to you because of your valuable insights and experience in [supporting family caregivers/the health sector/community building, etc.]. We are in discovery phase so your guidance today is vital to shaping our direction.

Permission to Record

We would like to record this session to capture your thoughts accurately. The transcript will be used internally for planning purposes and not shared. Is this OK with you? [If not, note that the interviewers will take notes instead.]

Engagement Promise

Your input will help guide the strategy development. Comments and quotes may be used to support our work but will remain anonymous. Please be open and share your thoughts.

Interview Record

- Date:
- Participants:
- Interviewer(s):

Interview Questions

1. **Current Focus and Priorities**
    - Can you tell me about your organization's interest, efforts and activities related to supporting family caregivers?

2. **Successes and Challenges**
   - What successes have you had in this work, and what challenges have you faced?
3. **Collaborative Partners**
   - Which sectors, groups, or organizations do you partner with to support family caregivers.
   - Which sectors, groups, or organizations do you think are critical for us to engage with? Why?
4. **Impact on Your Work**
   - How might a Caregiver Strategy and Action Plan support your efforts?
   - What do you hope it achieves specifically?
5. **Involvement Moving Forward**
   - How would you like to be involved as we move forward? (e.g., part of the co-design team, working on specific strategies, being kept informed, re-engagement)

Closing and Check-In

We have asked you lots of questions,

- Is there anything you would like to add or clarify?
- Do you have any final advice as we continue this journey?

Wrap-Up

Thank you for your time and valuable insights. Your contributions are essential to shaping a better future for family caregivers. It has been a pleasure speaking with you.

Supplementary File S3: Distinctive angles by cohort (what each brings)

- **Indigenous caregiving:** Wahkohtowin/extended kin; Indigenous navigator roles; dual-jurisdiction fixes (NIHB–provincial alignments); decolonized paperwork; lockboxes/discreet med delivery; bereavement travel funds.
- **HR & national experts:** Employer Champions Table, sector-specific playbooks, align with psychological health & safety, and long-duration/episodic caregiving—not just crisis leaves.
- **Palliative care:** Caregiver flag + Carer Support Needs Assessment Tool (CSNAT)-Intervention in workflows; warm-handoff requirement at discharge; practical respite (housekeeping/overnights); palliative approach literacy micro-learning; reinstate prefilled syringes; culturally safe end-of-life practices.
- **Children's disease-based orgs:** "Day-one" partnership with parents/caregivers; maintained, searchable resource directories; advocacy and communication toolkits; recognition that long-term caregiving spans school and health systems; refundable caregiver tax credits.
- **Adult disease-based orgs:** One accountable point of contact; privacy/consent consistency; sustainable funding for programs (beyond pilots).
- **Rural & community care:** Local, relationship-based navigation; transportation/internet barriers; inclusion of private/holistic providers and volunteers; crisis access to respite.
- **Seniors centres & Family and Community Social Services (FCSS):** Community hubs ready to be the local front door; respite-beds/empty lodge wings for respite; multi-year operating dollars over pilots; two-way referral agreements with health.
- **Transplant:** Donor/family triads; navigator/case manager across specialties; consented connections to peer support; travel/accommodation costs.
- **Primary care:** Use reform windows to hard-wire caregiver ID, triadic protocols, and navigator roles into team-based models; integrate 211/information exchange; include caregivers in emergency/transitional protocols; measure caregiver engagement.
- **Educators:** Put caregiver partnership into curricula, simulations, and accreditation; model in clinical placements.
- **Care of the Elderly & Geriatric medicine:** Triadic visits with separate space to speak privately to caregiver/patient; proactive placement planning; economic cases for policy; single named coordinator; refundable caregiver tax credits.
- **Home care & health care ops:** Rigid respite rules and "non-medical task" gaps are tipping points; formalize caregiver roles in workflows; let navigation expertise live with designated roles.
- **Geriatric psychiatry:** On-unit caregiver counselling/peer groups; consent toolkit for mental health; protect research/evaluation pathways through system changes.
